# Hospital Meetings

**Published:** 1896-08-08

**Authors:** 


### HOSPITAL MEETINGS.

### GROSVENOR HOSPITAL.

The annual meeting of the Grosvenor Hospital for Children and Women was held on the 31st ult. in the new out-patients' department, which has just been completed, and which stands on the site of a row of not prepossessing houses which faced Hide Place. Mr. A. J. Ram, the chairman of the Committee of Management, who presided, said that the meeting was this year little more than a formality, as it was overshadowed by the other very important events in the history of the hospital which had happened or were about to happen. As those present had no doubt noticed, the site for the new hospital was being prepared, and the ceremony of laying the foundation stone of the new building would take place very shortly. In fact, he hoped that in twelve months' time the new building would be erected. At the present time they had a balance on their building account, after paying for the out-patients' department, of £9,700, and they had entered into contracts for the hospital itself amounting to £8,632. Their income and expenditure account, however, was not in so flourishing a condition, for last year they had spent £1,615, whilst the income only amounted to £1,434. Therefore, had it not been for a legacy of £500, they would have had a deficiency of about £180. A resolution was passed reducing the qualification for a vice-president from £500 to £100. The ordinary routine matters, *i.e.*, the re-election of officers and the passing of votes of thanks, completed the business of the meeting.

